# Supplementary material for: How self-states help: Observing the embodiment of self-states through nonverbal behavior
Source: PLoS One. 2024 Mar 29;19(3):e0300682. doi: 10.1371/journal.pone.0300682 (PMC10980216; doi:10.1371/journal.pone.0300682)
Supplement: S1 Appendix — (PDF) [file pone.0300682.s001.pdf]

How self-states help:
Observing the embodiment of self-states through nonverbal behavior

- Supporting information -

Isabelle Engel[1], Maja Dshemuchadse[2], Caroline Surrey[1], Leander Roos[1], Philipp Kanske[1], Stefan Scherbaum[1]

[1] Department of Psychology, TUD Dresden Technical University, Dresden, Germany

[2] Department of Social Sciences, Hochschule Zittau-Görlitz, Görlitz, Germany

Corresponding author:
E-mail: Stefan.Scherbaum@tu-dresden.de

S1 Appendix - Manual of the Self-Constellation Method.

Self-state constellation method (duration: max. 90 min.)

1) Coaching assignment: conversational setting on chairs

- Inquire the decision topic brought by the coachee
- Write down the concrete decision options on yellow prompt cards (2-3), making sure that they are real options (no „non-options“).

2) Identification and prioritization of self-states: conversational setting on chairs

- Collect states, give each state a name (coachee) and write it down on white prompt cards (coach), paying attention to clarity and rewording negative names together.
    - o What states of you are calling out when you think about the decision options?
    - o What states do your parents/friends/partner know, who might be related to the decision?
- Select the states relevant to the decision (and combine linked states if necessary) so that the number of self-states becomes manageable (3-5)

3) Analogue work with self-states in the room: standing/walking in the room (coach stands outside the area with prompt cards - eye contact to coachee)

- Prompt cards are distributed around the room by coachee (options and states)
- Let the coachee look at the situation from a neutral position outside the cards:
    - o What do you notice when you see it like that?
- Round 1: Lead coachee to each state (coachee chooses order, coachee steps directly on card) and ask:
    - o You are [state name]. Please take a deep breath and feel inside yourself! What sensations/feelings do you perceive? How do you feel here? What thoughts are going through your mind? What do you stand for?
- Transition between states via neutral position
- Round 2: Guide coachee again to all states (same order if possible) and ask:
    - o You're [state name]. How are you doing here in your team?
    - o What is important to you regarding the options of the decision?
- In Round 3, Coach leads coachee to specific states and asks deeper questions:

  - Needs of the states: What is your mission for [coachee name]. Is there anything that could support you in this purpose?
  - Conflicts between the states: What might [coachee name] need the other state(s) for? What do you like about the other state(s)?
  - Appreciation/acceptance of all states: What are you important for / In which period were you particularly important for [coachee name]? And what do you need to feel better here and now with the other states?
  - Balance between states/ideas for compromise: What would you need to be okay with the decision option that you wouldn't choose for yourself?
  - New ideas of the states with regard to the decision
- At the end, Coachee again looks at his states from the outside: What has changed? Which feelings/thoughts are there now?

4) Conclusion

- Short conclusion in the discussion setting: How was it? Do you need anything else?
  - If the coachee feels that the decision is clear: ask him to think about it again calmly.
  - If the coachee feels that the decision is still unclear: ask him to endure and see what happens.
