# Supplementary material for: How self-states help: Observing the embodiment of self-states through nonverbal behavior
Source: PLoS One. 2024 Mar 29;19(3):e0300682. doi: 10.1371/journal.pone.0300682 (PMC10980216; doi:10.1371/journal.pone.0300682)
Supplement: S2 Appendix — (PDF) [file pone.0300682.s002.pdf]

How self-states help:
Observing the embodiment of self-states through nonverbal behavior

- Supporting information -

Isabelle Engel[1], Maja Dshemuchadse[2], Caroline Surrey[1], Leander Roos[1], Philipp Kanske[1], Stefan Scherbaum[1]

[1] Department of Psychology, TUD Dresden Technical University, Dresden, Germany

[2] Department of Social Sciences, Hochschule Zittau-Görlitz, Görlitz, Germany

Corresponding author:
E-mail: Stefan.Scherbaum@tu-dresden.de

S2 Appendix - Video Clip Selection.

Preselection

Out of 37 coachings, we selected numbers 2,3,9,15,19,22,26,28,31 and 34 according to the criteria below and renamed them 1-10 for better readability.

Visual Preselection:

1. Overview of coaching contours - How are the coach and coachee located in the room?
2. Is it possible (without disproportionate effort) to extract the coachee from the videos without the coach being visible?
3. Are there two frontal camera perspectives?

Acoustic Preselection:

1. Randomly listened to ten 10-second excerpts (only segments where the coachee is currently standing on a self-state)
2. Coachee is talking mainly from the perspective of the self-state (+) vs. talking about the self-state (-)

Preselection of Self-States:

1. At least 2 frontal camera perspectives of the state
2. Visual analysis of the spatial distribution of the states across the room (exclusion of the entire coaching session if states were swapped during the coaching)

Selection of the Self-States:

1. States at the poles of the constellation were selected first
2. Corrections (only applied, if thereby a state from the same pole could be replaced):

   - If two states were equally suitable: Decision in favor of the state that has more runs in the protocol (reasoning: indication of importance).

   - Correction in favor of the coachee questionnaire (states mentioned on the questions "best empathize" and "strongest emotion")

Selection of Short Video Clips

Two types of video clips were used in the rating study: video clips showing the states and a baseline video clip of the coachee before the intervention.

Selection of the 15s Video Clips Based on Two Criteria:

- Episodes in which the coachee moves a lot
- Episodes in which the coachee monologues (i.e., speaks as freely as possible without being interrupted by interactions with the coach)
- Selection of four 15s video clips based on the aforementioned criteria
    - Generally, two clips each from the first and second run of each state
    - In exceptional cases, when there were no clips in the second run that met the above criteria, selection of additional clips from the first or third run

Selection of the 6s Baseline Video Clips:

- Excerpts that were recorded before the actual states work began where the coachee
    - Was not actively engaged in a dialogue with the coach
    - Was not bending down to lay out prompt cards
    - Was not covered by the coach
